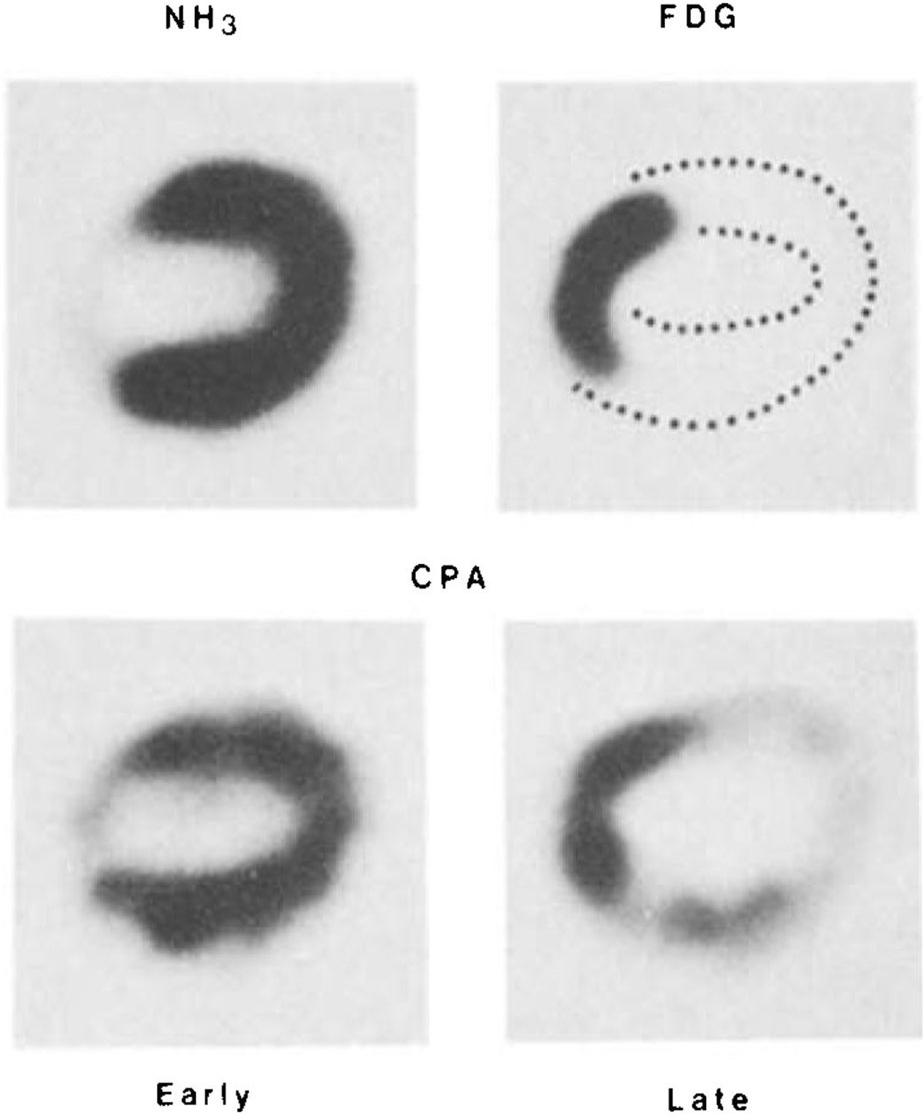# Markus Schwaiger, MD (Born 1950)

**DOI:** 10.1007/s12350-021-02765-w

**Published:** 2021-08-13

**Authors:** Frank M. Bengel, History Corner

**Affiliations:** Department of Nuclear Medicine, Hannover Medical School, Carl-Neuberg-Str. 1, 30625 Hannover, Germany

**Figure:**
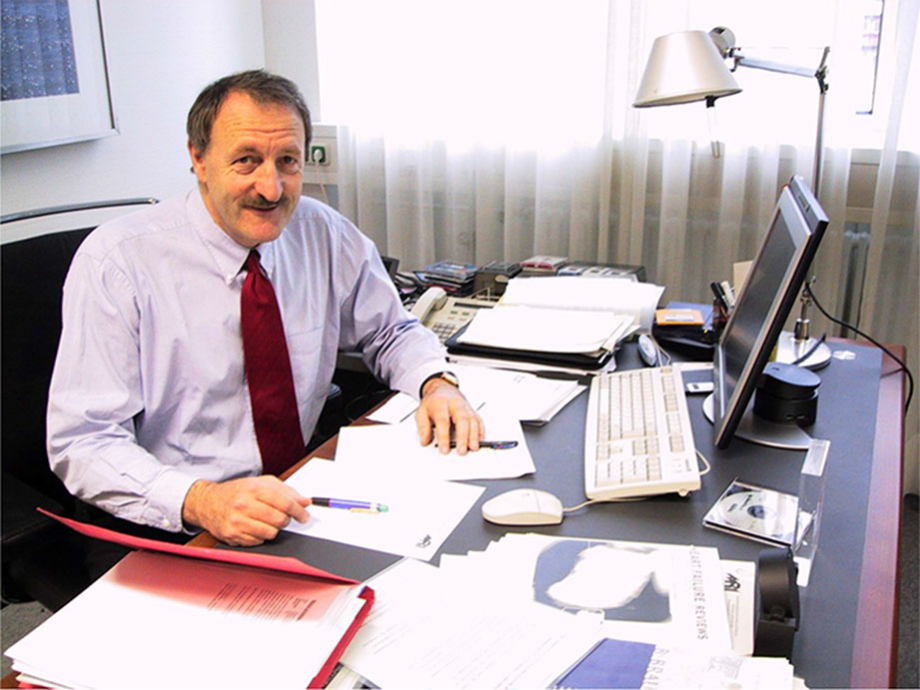
Markus Schwaiger, Chair of the Department of Nuclear Medicine at TU Munich (2002)

In the early days of coronary revascularization, there was a firm belief that chronically impaired contractile function in patients with extensive coronary artery disease was caused by irreversible myocardial damage. Besides clinical observations of functional recovery after revascularization, studies of myocardial blood flow and metabolism were a key to change this perception. Myocardial hibernation and stunning were identified as ischemia-induced, reversible states of contractile dysfunction that are amenable to revascularization. Markus Schwaiger published his pioneering experimental work in this regard in 1985.^1^ In a complex canine model, he employed serial positron emission tomography (PET) to show that the response to ischemia included sustained metabolic alterations. Increased regional uptake of the glucose analog F-18 fluorodeoxyglucose (FDG) and slow turnover of the fatty acid C-11 palmitate (CPA) suggested a metabolic switch from fatty acids to glucose as the substrate. This pattern was indicative of reversible injury and subsequent functional recovery. Soon after this preclinical work, clinical studies would emerge using PET for the assessment of myocardial viability in patients with left ventricular dysfunction, in order to predict functional recovery and clinical benefit from revascularization.^2^

This important research, early in his career, already displayed all the representative features of Markus Schwaiger’s subsequent profound and sustained scientific contribution to nuclear cardiology: Elegant and unique combinations of novel quantitative methods and radiotracers; a focus on molecular mechanisms; a translational approach including both preclinical and clinical aspects; and an emphasis on the creation of novel diagnostic and image-guided therapeutic paradigms. Those recurring characteristics of his research positioned him at the vanguard of the development of noninvasive cardiovascular imaging for several decades.

Markus Schwaiger was born on March 4, 1950, in the Bavarian capital Munich, where he grew up and went to school. His grandfather was a professor at the Technical University of Munich and his uncle was a surgeon in Freiburg. Both helped to stimulate his interest in the field of medicine, so that he moved from Munich to Berlin in 1969 to study medicine at the free university, where he graduated in 1975. He returned to Munich for his internship in internal medicine, which he briefly interrupted for an adventurous stint to the Hospital Andino of Coina in the Mountains of Peru, joined ,as always, by his wife Mechthild, also an MD, who he had met during medical school.

In Germany, a specific thesis is required to obtain the title of medical doctor (“Dr. med.”). Commonly, this is the first time when students or interns conduct a research project of their own, and it often defines the entry point into an academic career. For his MD thesis, which he completed in 1977, Markus Schwaiger chose to work on vectorcardiography. This was a first step into the direction of multi-dimensional noninvasive assessment of cardiac disease. In 1978, having obtained funding by the German Research Foundation, he decided to intensify his research experience and went to the University of Cincinnati as a postdoctoral fellow. In the Division of Physiology and Cardiology, he conducted research on the vascular effects of histamine, using an animal model of mesenteric ischemia. It was there that he learned to employ such disease-simulating experimental models for the introduction and validation of novel methodology. This early experience helped to shape a translational mindset, which became the foundation of his scientific career. The use of experimental aspects of imaging for the development of clinical applications would later emerge as a characteristic hallmark of many of his projects. He returned from Cincinnati to Munich a year later and started as a resident in internal medicine at the German Heart Center (after briefly considering cardiac surgery as an alternative pathway).

The open-minded, rapidly developing academic environment of the USA had a stimulating and profound impact on him, so that he decided to return after 3 years of resident training in Germany. He interviewed at various US universities and in the end chose to go to California. He would later describe his decision as mainly “driven by the prospects of outdoor sports and quality of life”—but his own mindset, the charismatic mentorship by Heinz Schelbert, and the stimulating and world-leading positron emission tomography program at UCLA evidently supported his focus on a rapidly evolving scientific and academic career. In his 6 years as a fellow and assistant professor at UCLA, he successfully used PET imaging in many projects, contributing to completely novel insights into the regulation of myocardial flow dynamics and substrate utilization.

When Dave Kuhl, the director of nuclear medicine and a leading pioneer in tomographic imaging, moved from UCLA to University of Michigan in 1986, he asked Markus Schwaiger to join him and lead the nuclear cardiology & cardiac PET program in Ann Arbor. The move turned out to be a tremendous success and nuclear cardiology flourished at University of Michigan. Many national and international researchers would join Markus Schwaiger’s group in Ann Arbor, and many would later emerge as leaders of their own in the fields of nuclear cardiology and nuclear medicine. While continuing his research with PET to define determinants of myocardial flow dynamics and substrate metabolism, Markus Schwaiger also expanded his work toward the field of SPECT and novel tracers, with the goal of promoting the clinical use of noninvasive viability and ischemia testing. Importantly, he also established collaboration with Donald Wieland as the leading expert in radiolabeling of catecholamines. This led to a novel and very successful program in PET imaging of the cardiac sympathetic nervous system, which provided unique insights into the role of autonomic innervation in heart failure, arrhythmia, and diabetes. Also, for the first time, the process of sympathetic reinnervation of the transplanted heart was captured by means of quantitative noninvasive imaging.^3^

After having moved up the ranks in Michigan, where he earned full professor status in 1991, Markus Schwaiger had the opportunity to return to his birthplace in Munich / Germany in 1993, as the director of the local Department of Nuclear Medicine at Klinikum rechts der Isar of the Technical University of Munich. Building upon the success of his Michigan program, Markus Schwaiger continued to expand his endeavor in cardiovascular imaging. He established successful clinical cardiac PET and SPECT services along with a research program that continued to benefit from an influx of international postdoctoral researchers. The program was nourished by novel molecular-targeted tracers such as integrin ligands, which emerged from the successful radiopharmaceutical chemistry group of his department. Additionally, methodological advances were adopted, including the in-house development of the analysis software tool “Munich Heart”, the development and installation of preclinical small animal imaging techniques, and the development of a clinical cardiac magnetic resonance imaging unit that further expanded the multi-modal, multi-parametric research environment of his department.

As the chair of a large nuclear medicine department, Markus Schwaiger expanded his research beyond cardiovascular imaging, in order to exploit the cross-sectional nature of molecular imaging and contribute to the increasing success and penetration of the “theranostic” principle (which integrates diagnostic and therapeutic versions of the same radioligand). Accordingly, oncology emerged as a second main pillar of his program in Munich, which would soon outgrow cardiovascular imaging applications. He obtained several major grants for oncologic imaging and image-guided therapy, including an Advanced Grant by the European Research Council, a full-scale interdisciplinary Collaborative Research Center by the German Research Foundation, and various others. Importantly, this broad success in radiotracer applications, along with the existing expertise in magnetic resonance and integrative multi-modal imaging, turned out to be a natural foundation for his pioneering role in integrated PET-MR hybrid imaging. In 2010, his department was the first installation site worldwide of the integrated whole-body PET-MR system, Siemens Biograph mMR, triggering many key contributions to the evolution of this disruptive technology.^4^ Later, the clinical device would be amended by additional preclinical PET & MR systems and by hyperpolarizer capabilities for targeted MR contrast agents.

The profound success of his nuclear medicine and molecular imaging program, along with his visionary networking and managerial skills, destined Markus Schwaiger to take on superordinate university leadership roles. Starting as an associate dean for student affairs in 1999, he would become vice dean and then was the dean of the medical school at the Technical University of Munich for 8 years, steering medical research and teaching of his entire university in parallel to chairing his nuclear medicine department. In 2017, Markus Schwaiger retired as the chair of the Department of Nuclear Medicine at TU Munich, which he had developed into one of the leading nuclear medicine facilities of the world. Ten of his former trainees have moved on to become chairs of nuclear medicine departments in German university hospitals, continuing his legacy. He published more than 800 peer-reviewed papers in international scientific journals and more than 100 book chapters. As of early 2021, he has an h-index of over 125 according to Web of Science. He obtained the Paul C Aebersold Award and the Hermann Blumgart Award of the Society of Nuclear Medicine and Molecular Imaging, the ESMI Award of the European Society of Molecular Imaging, and the Georg von Hevesy Medal of the German Society of Nuclear Medicine. He is a member of the German National Academy of Sciences “Leopoldina” and a member of the Bavarian Academy of Sciences. For his national services, he received the German Federal Cross of Merit and the Bavarian Maximilian Medal for Science and Art. He also received an honorary doctorate from the University of Varna (Dr. med. h.c.) and the Heinz Maier Leibnitz Medal of the Technical University of Munich.

His retirement from nuclear medicine in 2017, however, would not be equal to his retirement from academic medicine. In late 2016, he had already taken on a new leading role, this time as the medical director and CEO of his university hospital, the Klinikum rechts der Isar in Munich. For a term of 5 years, he would lead this ship through the economic and political challenges of German academic medicine, and most recently through the unforeseeable demands of the COVID pandemia. In July 2021, he finally went into “real” retirement. He is still active as a sought-after speaker and advisor, but he also enjoys quality time in his summer residency by the “Bavarian Sea” (the lake “Chiemsee”), and he will continue to pursue his passion of alpine skiing. While he is now looking forward to spending more time with his wife Mechthild, his four children, and his increasing number of grandchildren, he will certainly be proud when he looks at his large number of “academic children”, who are spread all over the world in the fields of cardiovascular imaging and nuclear medicine.

ICONIC PET IMAGES of dog heart, early and late after ischemia and reperfusion

Myocardial perfusion (N-13 Ammonia,NH_3_), Glucose metabolism (F-18 FDG), and Fatty Acid turnover (C-11 Palmitate, CPA). *Reprinted from reference 1, with permission.*